# Plasma exosomal miRNAs-based prognosis in metastatic kidney cancer

**DOI:** 10.18632/oncotarget.19476

**Published:** 2017-07-22

**Authors:** Meijun Du, Karthik V. Giridhar, Yijun Tian, Michael R. Tschannen, Jing Zhu, Chiang-Ching Huang, Deepak Kilari, Manish Kohli, Liang Wang

**Affiliations:** ^1^ Department of Pathology and MCW Cancer Center, Medical College of Wisconsin, Milwaukee, WI, USA; ^2^ Department of Oncology, Mayo Clinic, Rochester, MN, USA; ^3^ Department of Oncology, Tongji Hospital of Tongji Medical College, Huazhong University of Science and Technology, Wuhan, P.R. China; ^4^ Human and Molecular Genetics Center, Medical College of Wisconsin, Milwaukee, WI, USA; ^5^ Department of Biostatistics, University of Wisconsin, Milwaukee, WI, USA; ^6^ Department of Oncology, Medical College of Wisconsin and Milwaukee VA Medical Center, Milwaukee, WI, USA

**Keywords:** metastatic renal cell cancer, exosomal miRNA, overall survival, biomarker

## Abstract

Plasma exosomal miRNAs were evaluated for prognosis in an initial set of 44 metastatic renal cell cancer (mRCC) patients by RNA sequencing. Among ∼3.49 million mappable reads per patient, miRNAs accounted for 93.1% of the mapped RNAs. 227 miRNAs with high abundance were selected for survival analysis. Cox regression analysis identified association of 6 miRNAs with overall survival (OS) (P<0.01, False discovery rate (FDR) < 0.3). Five of the associated miRNAs were quantified in an independent follow-up cohort of 65 mRCC patients by TaqMan-based miRNA assays. Kaplan-Meier analysis confirmed the significant OS association of three miRs; miR-let-7i-5p (P=0.018, HR=0.49, 95% CI=0.21-0.84), miR-26a-1-3p (P=0.025, HR=0.43, 95% CI=0.10-0.84) and miR-615-3p (P=0.0007, HR=0.36, 95% CI=0.11-0.54). A multivariate analysis of miR-let-7i-5p with the clinical factor-based Memorial Sloan-Kettering Cancer Center (MSKCC) score improved survival prediction from an area under the curve (AUC) of 0.58 for MSKCC score to an average AUC of 0.64 across 48-month follow-up time. The multivariate model was able to define a high-risk group with median survival of 14 months and low risk group of 39 months (P=0.0002, HR=3.43, 95%CI, 2.73-24.15). Further validation of miRNA-based prognostic biomarkers are needed to improve current clinic-pathologic based prognostic models in patients with mRCC.

## INTRODUCTION

Approximately 30% of renal cell carcinoma (RCC) patients have metastases at the time of initial diagnosis, and in patients who are initially treated for localized stage disease, metastasis occurs in 30-50% following complete resection of the primary tumor [1, 2]. The clinical course of metastatic RCC (mRCC) is heterogeneous, ranging from aggressive disease (which can be fatal within months), to a more indolent progression that can be monitored for years before treatment. Several new treatments have been approved recently in metastatic stage [3] and determining prognosis is clinically relevant for initiating systemic treatments especially in patients with average to poor prognosis.

In clinical practice the most common scoring system used for prognostication is the Memorial Sloan Kettering Cancer Center (MSKCC) prognostic score [4, 5]. This is based on clinical factors (serum calcium, hemoglobin, LDH levels, performance status and presence or absence of nephrectomy), and does not include sensitive or specific genomic biomarkers that may reflect underlying stage-specific tumor biology associated with disease progression [6, 7]. Prognostic biomarkers provide information about survival, regardless of therapy. Identification of prognostic biomarkers is also often the first step which gives insight about a potential biological pathway to target and also to develop efficacy-based factors for that therapeutic intervention, i.e. a predictive biomarker [8, 9]. New molecular prognostic biomarkers in RCC that reflect genetic changes may enhance the prediction of survival outcomes including aggressiveness [10–12]. However, in metastatic stage tissue-based prognostic markers are difficult to obtain unless metastases are biopsied. An alternative may be to identify genetic biomarkers such as miRNAs in blood and urine that could be assessed noninvasively to improve prognostication in mRCC.

miRNAs are a class of small, single stranded, non-coding RNA molecules of 19–24 nucleotides [13] that play important roles in numerous cellular process including proliferation, apoptosis, metabolism, and differentiation [14]. miRNAs remain stable and intact in serum/plasma samples, likely due to binding to RNA binding proteins or lipoproteins, or being embedded inside circulating microvesicles. Exosomes in the form of microvesicles are small sized (30–100 nm) membrane vesicles which are released into the extracellular environment and have the ability to remain stable in serum [15, 16]. These blood-based exosomes can be sampled easily, which makes them particularly attractive for clinical applications including diagnosis, prognosis [17] and for monitoring of disease or treatments by serially and noninvasively performing repeated measurements over time. In this study, we performed a comprehensive expression profiling analysis of plasma exosome miRNAs using a RNA-sequencing approach in mRCC patients and investigated for association of these miRNAs with OS.

## RESULTS

### Clinical characterization of patient samples

Clinical and pathological characteristics are recorded and summarized in Table 1. In the screening cohort (N=44), 40/44 patients had clear cell renal carcinoma and most samples were obtained before any systemic therapy (23/44) or after one systemic therapy (10/44). The median followup time for this cohort was 3.76 years, during which time 26 patients had died. An independent follow-up cohort of mRCC patients (N=65) was used to test candidate miRNAs identified after RNA sequencing. Study specimens from blood were obtained prior to any systemic therapy in 40/65 and after one systemic therapy in 19/65. The most common histology in this cohort was clear cell carcinoma (52/65), followed by papillary (6/65), and chromophobe (2/65), while remaining 5 (5/65) were unspecified. The median followup time of this cohort was 2.85 years, during which time 41/65 patients had died. Of the patients where treatment-specific follow up was available (32/44 in screening cohort, 50/65 in validation cohort), the systemic treatments are described in Table 1.

**Table 1:** Clinical characteristics of RCC patients in both cohorts

Patients characteristics		Screening cohort (RNA seq)	Validation cohort (qPCR)
		n = 44	n = 65
**Age (median, IQ)**		70.2 (61.9, 76.9)	64.6 (60.6, 74.6)
**Gender**	Male (n)	35	48
	Female (n)	9	17
**Histology**	Clear Cell	40	52
	Chromophobe		2
	Papillary	2	6
	Unspecified	2	5
**Furhman grade**	G1	3	
	G2	13	21
	G3	10	22
	G4	13	11
	Unspecified	5	11
**Sarcomatoid differentiation at diagnosis**	Present	7	3
	Absent	37	62
**Clinical stage at initial diagnosis**	I	10	7
	II	7	8
	III	8	20
	IV	16	29
	Unspecified	3	1
**Tumor stage**	T1	9	9
	T2	4	8
	T3	18	31
	T4	2	8
	TX	11	9
**N stage**	N0	18	35
	N1	9	10
	Nx	17	20
**M stage**	M0	25	34
	M1	17	30
	MX	2	1
**Median time (years) from diagnosis to metastases for initial M0 (range)**		2.47 (0.13, 22.15)	2.61 (0.09, 17.54)
**MSKCC prognostic category at the time of metastases**	1	20	38
	2	18	16
	3	4	11
	Missing	2	0
**Median time (years) to death or last follow up (range)**		3.76 (0.35, 14.45)	2.85 (0.11, 17.61)
**Number of deaths**		26	41
**Total lines of systemic therapy**	0 (active monitoring)	5	4
	1	12	20
	>=2	23	28
	Unknown	4	13
**Systemic therapy (given during any time of follow up)**		**n = 32**	**n = 50**
Sunitinib		21	28
Sunitinib, 1st line		19	22
Sorafenib		4	3
Pazopanib		18	27
Axitinib		5	13
Everolimus		7	13
Temsirolimus		10	17
Pembrolizumab		0	1
Nivolumab		4	3
Interferon or IL-2		1	2
Bevacizumab		2	2
Bevacizumab + IFN		0	2
Chemotherapy		0	2
Other		2	2
**Sample collection**		**n=44**	**n=65**
Before any systemic treatment		23	40
After systemic therapy		21	25
After one systemic therapy		10	19

### Overall quality of exosomal RNAs and sequencing libraries

We determined the quality and quantity of the extracted exosomal miRNAs with small RNA Chip in the initial cohort of 44 patients. Our data showed that the RNA sizes ranged from 16–32 nucleotides (nt) with a peak at 19-22 bp. We also observed another size range from 38 to 60 bp with a peak at 45 bp, representing other RNA species in the plasma exosome (Supplementary Figure 1A). The average RNA yield for each sample was ∼20 ng (ranged from 5 to 44 ng). We applied High Sensitivity DNA Chips to examine the quality and quantity of the RNA sequencing library. Supplementary Figure 1B showed typical results from a miRNA library with a peak band of 147 bp, corresponding to 21 bp miRNA plus adaptors. Another peak at 125 bp was estimated to be adaptor-adaptor ligation, as it was the same size as the ddH2O negative control. The overall peak band of these RNA libraries was from 143-158 bp. The target bands at 140-160 bp in the pooled libraries were recovered for sequencing (Supplementary Figure 1C).

### Plasma exosomal RNA profiling

**Figure 1:**
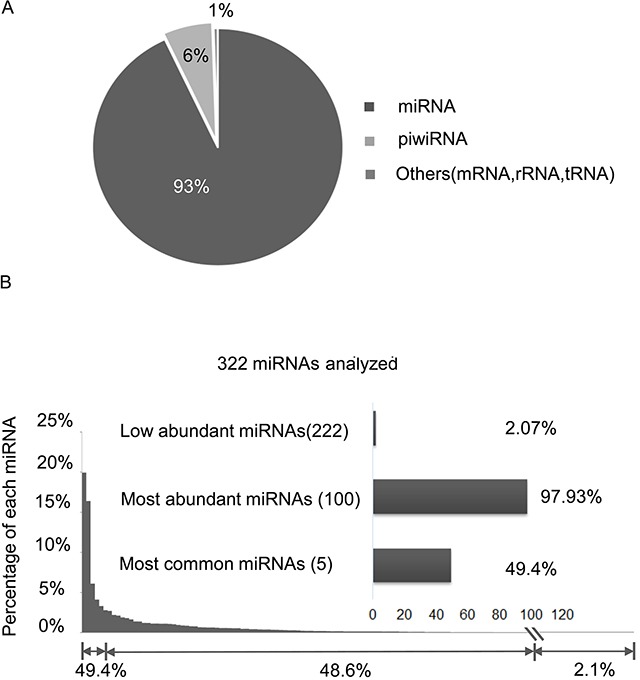
Statistical summary of RNA species detected by RNA-seq across 44 RCC libraries **(A)** RNA species detected in the exosomal RNA libraries. **(B)** Percentage (sorted from high to low) of each of the 322 mature miRNAs in all mapped miRNA reads.

From 44 exosomal RNA sequencing libraries, we received an average of 8.77 million raw reads per library. Of these, 44.7% (3.49 million reads) were mapped to miRNAs, piwi RNAs and other RNAs including mRNA, rRNA and tRNA. Among the mapped reads, mature miRNAs were the most common with an average of 3,489,544 reads (93.08%), followed by piwi-interacting RNA with 239,229 (6.39%), other RNAs (mRNA, tRNA, rRNA etc.) with 19,964 (0.53%) (Figure 1A, Supplementary Table 1). Herein we report our analysis on 322 common miRNAs with >8 RPMs (reads per million mappable count) (Supplementary Table 2). Distribution of the 322 miRNAs was shown in Figure 1B. The five most common miRNAs (miR-128-3p, miR-99a-5p, miR-9-5p, miR-129-5p, and miR-22-3p) collectively accounted for 49.4% of all mappable miRNA sequences. The 100 most abundant miRNAs made up 97.93% of the detectable miRNA sequences; the remaining 222 low abundant miRNAs accounted for 2.07%. These results were similar to our previous study in other tumor types [18]. The raw sequencing data were deposited in the Gene Expression Omnibus database (accession number: GSE93175).

### Association of plasma exosomal miRNAs with overall survival in screening cohort

To identify survival-related exosomal RNAs in mRCC patients, we further excluded those miRNAs with log2 transformed read counts <5. The remaining 227 miRNAs were used for Cox regression analysis. Six miRNAs were detected to be associated with overall survival (OS) (p-value<0.01, False discovery rate (FDR)<0.3). These included miR-190b, miR 26a-1-3p, miR-let-7i-5p, miR-145-3p, miR-200-3p and miR-9-5p. Low expression of miR-let-7i-5p was associated with poor OS (HR=0.584, p value=0.0060, 95% CI: 0.398-0.857). Table 2 lists all miRNAs significantly associated with OS. Clinical factors such as age, sex were not correlated with miRNAs expression level.

**Table 2:** miRNAs associated with overall survival in plasma in the screening cohort

RNA Name	Hazard ratio	Lower limit CI	Upper limit CI	p-value	FDR
hsa-miR-190b	0.4609	0.3040	0.6890	0.0003	0.0391
hsa-miR-26a-1-3p	0.4691	0.3078	0.7148	0.0004	0.0391
hsa-miR-145-3p	0.5258	0.3588	0.7706	0.0010	0.0597
hsa-miR-200a-3p	0.5222	0.3474	0.7849	0.0018	0.0814
hsa-let-7i-5p	0.5848	0.3989	0.8573	0.0060	0.2188
hsa-miR-9-5p	0.5507	0.3504	0.8656	0.0097	0.2963
hsa-miR-615-3p	0.5359	0.2965	0.9684	0.0388	0.4281

### miR-127-3p as the best endogenous normalizer

To identify potential miRNA references for target miRNAs quantification, we first analyzed the 322 miRNA expression profiles from the 44 exosomal RNA sequencing libraries and selected the top 50 miRNAs with the smallest CV(coefficient of variation). We then applied RefFinder to identify the most stably expressed RNAs (Supplementary Table 3). Based on their expression levels and stability, the top three most stable miRNAs (miR-127-3p, miR-532-3p and 27a-3p) were considered as the reference candidates (Supplementary Figure 2A). We also selected miRNA-30a-5p as candidate internal controls, as it was previously reported to be the best endogenous normalizer [19]. To confirm the best endogenous RNA references, we examined candidate miRNAs using the TaqMan qRT-PCR assay in the follow-up cohort. The results showed that miR-127-3p was the most stably expressed and was used as the endogenous normalizer in this study (Supplementary Figure 2B).

### Association of miR-26a-1-3p, miR-let-7i and miR-615-3p with OS in the validation cohort

**Figure 2:**
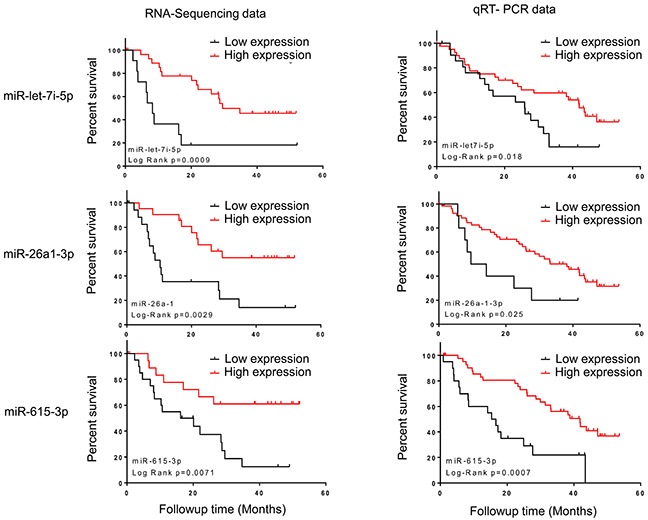
Kaplan–Meier curves showing the association of miRNA abundance and OS in plasma of mRCC patients Left panel: sequencing data. Right panel: qRT-PCR data, the p values were computed by log-rank test. miR-let-7i-5p, miR-26a-1-3p and miR-615-3p are shown.

To validate the association of miRNA expression levels with OS, we examined isoform composition of 6 statistically significant candidate miRNAs and selected 4 miRNAs for further validation, including miR-26a-1-3p, miR-let-7i-5p, miR-9-5p, and miR-190b. Additionally, miR-615-3p was selected due to its potential prognostic value in mantle-cell lymphoma [20]. We measured the expression of each selected miRNA by TaqMan qRT-PCR in an independent mRCC cohort. After normalizing expression values of these targets to the internal control (miR-127-3p), Kaplan-Meier analysis confirmed the significant association of miR-let-7i-5p (P=0.018, HR=0.49, 95% CI=0.21-0.84), miR-26a-1-3p (P=0.025, HR=0.43, 95% CI=0.10-0.84) and miR-615-3p (P=0.0007, HR=0.36, 95% CI=0.11-0.54) with OS. These results were consistent with sequencing-based association with OS observed in the initial cohort (Figure 2A). At 30 month follow up the mortality rate for patients with lower expression of miR-let-7i-5p was 70%; and 80% for miR-615-3p and miR-26a-1-3p. Higher expression of these three miRNAs was associated with a mortality rate of less than 40% over the same follow up period (Figure 2B). The association of miR-9-5p and miR-190 with OS was not statistically significant (Supplementary Figure 3). No significant association was found between the levels of expression of miR-let-7i, miR-615-3p and miR-26a-1-3p with the clinical parameters of sex or age (Table 1). Moreover, no significant association of age, gender, tumor grade, stage at diagnosis, coagulative necrosis, or sarcomatoid differentiation were found with the OS in mRCC patients (Supplementary Table 4).

### Multivariate model for prognosis of mRCC

**Figure 3:**
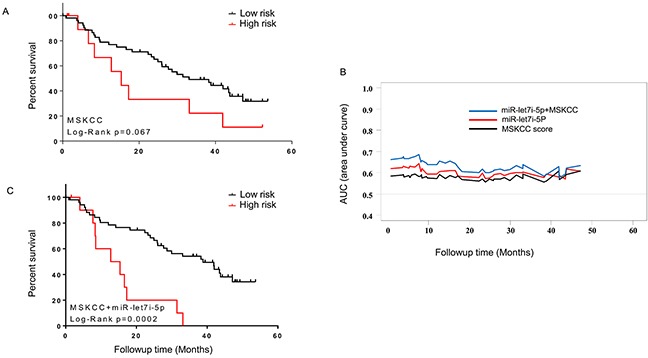
Multivariate prognostic model combining the expression of miR-let-7i and MSKCC score **(A)** Kaplan-Meier analysis showing association of MSKCC score with OS. **(B)** Time-dependent AUC analysis for 65 mRCC patients from the follow-up cohort. The multivariate model (miR-let-7i and MSKCC score) improved the survival prediction compared to miR-let-7i or MSKCC alone. **(C)** Kaplan-Meier analysis showing association of multivariate model (miR-let-7i expression and MSKCC score) with OS.

We first evaluated MSKCC prognostic score and its association with OS in the follow-up cohort and observed a borderline significance (HR=2.02, p-value=0.0675, 95% CI, 0.94-6.81 (Figure 3A). The MSKCC score alone showed a predictive performance of OS with AUC (average area under ROC curve)=0.58 (Figure 3B). We tested the predictive performance of the 3 validated miRNAs and observed only AUC value of miR-let-7i-5p (0.60) being higher than MSKCC score. In addition to Kaplan-Meier analysis, Cox regression model also revealed miR-let-7i-5p was significantly associated with OS (P=0.007, HR=0.566, 95% CI: 0.374-0.857). After adjusting for the MSKCC score, miR-let-7i-5p remained significantly associated with OS (P=0.006, HR=0.566). To examine whether incorporation of miR-let-7i into MKCC score could improve the prediction performance, we combined miR-let-7i-5p with the MSKCC score and evaluated time-dependent AUC in the follow up cohort. Specifically, the risk value was a linear combination of the miR-let-7i-5p expression levels and MSKCC score, weighted by their coefficients from the univariate Cox regression analysis. The multivariate analysis showed that integration of miR-let-7i-5p into MSKCC score improved its predictive performance. The multivariate model had an average AUC of 0.64 across 48-month follow-up time while the MSKCC score alone showed an average AUC of 0.58 in the same period (Figure 3B). The multivariate model (miR-let-7i-5p and MSKCC score) was positively associated with OS (P=0.0002, HR=3.43, 95% CI: 2.73-24.15). Further analysis demonstrated that high risk group had a median survival of 14 months while low risk group had a median survival of 39 months (Figure 3C).

## DISCUSSION

In this study, RNA sequencing of plasma exosomal miRNAs revealed potential prognostic biomarkers in patients with mRCC. Although several clinical and pathological determinants of prognosis (e.g. MSKCC prognostic score, TNM stage, Fuhrman grading, performance status) have been developed for use in current clinical practice, the ability to accurately predict survival outcome has limitations as these markers do not reflect underlyng genomic drivers of biology [4, 21]. Genetic prognostic biomarkers are likely to have higher sensitivity and specificity as they more accurately reflect tumor biological characteristics in mRCC stage and possibly account for tumor heterogeneity more directly. miRNAs have been reported with potential uses as diagnostic biomarkers, subtyping tumor types, and predicting survival at the time of nephrectomy [22–29]. However, these studies examined miRNAs in cell lines or paired normal-tumor tissues. Tissue biopsies are invasive, more cumbersome, and carry the risk of complications for patients. Plasma samples, or liquid biopsies, provide an opportunity to identify prognostic, predictive, and monitoring biomarkers that can be more easily accessed and applied in clinical practice.

Previously, miRNA expression profiles have been used successfully to distinguish between malignant and non-malignant tissue, and have also been used to inform diagnosis and risk of cancer recurrence [30–33]. Circulating miRNAs are also used as diagnostic biomarkers in RCC, helping to distinguish cancer patients from healthy controls [34, 35]. Exosomal miRNA profiles from plasma have identified prognostic biomarkers in patients with advanced prostate cancer [19], esophageal squamous cell carcinoma [36], and pancreatic adenocarcinoma [37]. However, exosomal miRNA profiles in mRCC patients have not been reported. In this study by using RNA-seq we established a large-scale exosomal miRNA expression profile in mRCC patients and identified 6 miRNAs whose abundance levels in plasma were significantly associated with OS. Importantly, three of five selected miRNAs (miR-26a1-3p, miR-let-7i-5p and miR-615-3p) were subsequently validated in an independent cohort. Furthermore, at the univariate level the prognostic ability of miR-let-7i-5p was observed to be better than MSKCC prognostic score. Most importantly, in our multivariate Cox regression model, the miR-let-7i-5p remains as an independent predictor for OS after adjusting for the MSKCC score. The multivariate model showed that combining miR-let-7i-5p with the MSKCC score enhanced the prognostic estimate for survival for this stage.

This is the first study that identifies and validates circulating exosomes-based miRNA as prognostic biomarkers of OS in mRCC. Our results are also consistent with previous studies [38, 39] in tissue biopsies, which described a decrease in the expression of the miR-26a and let-7 family (let-7b and let-7c) in highly aggressive primary metastatic tumors in clear cell RCC [38, 39]. Functionally, miRNA let-7's action as a tumor suppressor in RCC cell lines occurs by down-regulating C-myc and C-myc target genes [40]. The dysregulation of let-7b and let-7c may be involved in treatment resistance of RCC cells to 5-flouro-uracil (5-FU) by down-regulating Akt2 [39].

There are several limitations of our prediction model that need attention in future studies before it can be implemented as a clinical prognostic tool. First, we did not analyze the different isoforms of miRNA molecules from RNA sequencing data, which may lead to missing other relevant miRNA families, especially for the miRNAs with differences only at 5′- or 3′- end nucleotides. Another limitation is the selection of internal controls for qRT-PCR normalization to eliminate the input difference of the original cDNA template. Due to difficulty in quantifying exosome RNA yield and varying efficiency in reverse transcription, the cDNA input varies from sample to sample, possibly leading to bias when comparing different samples. This limitation was at least partially reduced by our extensive selection of miRNAs with the most stable abundance. Although miR-127-3p was stable in RCC patients, whether this miRNA can be used as a reference in normal plasma or other cancer samples need to be further validated. Additionally, while the majority of samples analyzed were clear cell RCC, 9.2% were non-clear cell subtype (Table 1). The small sample size of non-clear cell subtypes prohibited any separate analysis, therefore further analysis in a larger sample set with attention to a specific histology will remain an important goal for assessing this tool for this patient subset. Finally, metastatic kidney cancer patients have at least twelve drug treatments available. When developing prognostic markers for metastatic stage patients, it is important to consider downstream systemic treatments in larger datasets.

In summary, our study identifies multiple plasma exosomal miRNAs showing association with survival in mRCC stage patients. The examination of exosomal miRNA from blood is a non-invasive and attractive approach and can be applied in a routine clinical setting by performing qRT-PCR-based miRNA expression analysis.

## MATERIALS AND METHODS

### Patient samples

A total of 109 advanced mRCC plasma samples were collected in a prospective cohort at Mayo Clinic. Patients with mRCC were recruited between October 2011 and May 2015, and samples were collected before or during any line of systemic therapy. All participants gave written informed consent prior to study participation. Use of clinical sample cohorts in this study was approved by Institutional Review Boards at both Mayo Clinic and Medical College of Wisconsin. Plasma was collected, uniformly processed, and stored at −80°C before use, as previously described [41]. A screening cohort of mRCC plasma samples were used for exosome isolation, RNA extraction, and sequencing-based biomarker discovery. A follow-up cohort was used for the data validation by TaqMan quantitative real-time reverse transcription polymerase chain reaction (qRT-PCR).

### Exosome precipitation and RNA isolation

Exosomes were isolated using ExoQuick (System Biosciences, Mountain View, CA, USA) according to the manufacturer's instructions with minor modifications. Briefly, 250 μL of plasma was incubated with 250 μL thromboplastin D (Thermo Scientific, Middletown, VA, USA) for 15 min at 37°C. After centrifugation at 10,000 rpm for 5 min, the supernatant was mixed with 125 μL of ExoQuick solution. The mixture was kept at 4°C overnight and centrifuged at 1500 g for 30 min. The exosome pellet was dissolved in 25 μL 1 × PBS and RNA was extracted using miRNeasy Micro Kit (Cat# 217084, QIAGEN, Valencia, CA, USA). The 25 μL of exosome suspension was mixed with 700 μL QIAzol lysis buffer, and the mixture was processed according to the manufacturer's standard protocol. The extracted RNA was eluted with 15 μL of RNase-free water. The quantity and quality of the RNAs were determined by Agilent Bioanalyzer 2100 with a Small RNA Chip (Agilent Technologies, Santa Clara, CA, USA).

### RNA sequencing library preparation

The 44 RNA libraries were prepared following the instructions of the standard protocol (NEBNext Multiplex Small RNA Library Prep Set, NEB, Ipswich, MA, USA). For each library, 2 ng of miRNA was used as input RNA. Each library was prepared with a unique indexed primer (Index set1 and set2, NEB). The amplified libraries (DNA) were qualified by Agilent High Sensitivity DNA Chips (Agilent Technologies, Santa Clara, CA, USA). Twenty-two RNA samples were pooled with equal molar concentration. Two pooled libraries were resolved on a 5% Mini-Protean Precast Gels (Bio-Rad, Hercules, CA, USA). DNA fragments from 140–160 bp (the length of miRNA inserts plus the 3′ and 5′ adaptors) were recovered in 12 μL elution buffer (QIAGEN, Valencia, CA, USA). The indexed libraries were quality-checked and quantified by Agilent High Sensitivity DNA Chips. Ten μL of the pooled library at a final concentration of 2 nM were then sent to the Core Facility at Medical College of Wisconsin for 50 bp single read sequencing using Illumina HiSeq2000 DNA sequence analyzer.

### Sequencing data analysis

The RNA sequencing data (Fastq files) were mapped using software package (DNASTAR SeqMan NGen) against the human miRNA sequences downloaded from miRBase 21 (http://www.mirbase.org/) [42], piwiRNA sequences downloaded from piRNA-Bank Version 2 (http://pirnabank.ibab.ac.in) and the human genome reference sequences downloaded from the NCBI ftp site (ftp://ftp.ncbi.nlm.nih.gov). The miRNA profiles were normalized to RPM, which was based on the following formula: read counts of an individual miRNA/sum of read counts of all mappable miRNAs multiplied by 1 × 10^6^.

### Identification of endogenous references

The normalized sequencing data from 44 mRCC patients were first screened for the top 50 most stably expressed miRNAs with an overall CV <0.0265. The 50 miRNAs were further evaluated using the software Reffinder (http://fulxie.0fees.us), which integrates the currently available major computational programs (the comparative ΔCt, Genorm, and Normfinder) and generates the overall stability ranking [43]. In the follow-up cohort, raw CT values derived from real-time PCR were used to determine their stabilities.

### TaqMan quantitative RT-PCR

TaqMan-based miRNA assays were performed to measure the expression levels of candidate normalization controls (miR-127-3p; miR-532-5p; miR-27a-3p; miR-30a-5p) and target miRNAs (miR-26a-1-3p, miR-9-5p; miR-let-7i-5p;-miR-615-3p; and miRNA-190b) by using the plasma exosomal RNAs in the follow-up cohort, according to the manufacturer's instructions (Applied Biosystems, Life Technology, Waltham, MA, USA). 2 uL microRNA was reversely transcribed by using the TaqMan Advanced miRNA cDNA Synthesis Kit following standard protocol with 2 ng for most of the starting miRNAs (Applied Biosystems, Life Technology). PCR reaction was performed in triplicates and analyzed using BIO-RAD CFX384 Real-Time PCR Detection System. The relative abundance of miRNAs expression among these patients was determined by the ΔCT methods.

### Survival analysis

OS was determined from the date of plasma collection after metastases to the date of death or last follow-up. Association between exosomal miRNA levels and OS was assessed using Cox regression in Partek Genomics Suite 6. FDR was used to correct for multiple testing. Kaplan-Meier analysis and log-rank test were used for showing survival association (GraphPad Prism 6). The best cutoff values for survival prediction was determined by the software Cutoff Finder (http://molpath.charite.de/cutoff/). Time-dependent receiver operating characteristic (ROC) curve analysis was used to compare the predictive performance of the risk scores for the combined miRNAs and/or clinical variables. The AUC across 48-month follow-up time was used to evaluate survival prediction and AUC plots were created using the risksetAUC function in the R package: risksetROC (www.r-project.org).

## SUPPLEMENTARY MATERIALS FIGURES AND TABLES









## Abbreviations

mRCC: metastatic renal cell carcinoma
RCC: renal cell carcinoma
miRNAs: microRNAs
OS: overall survival
qRT-PCR: quantitative real-time reverse transcription polymerase chain reaction
MSKCC: The Memorial Sloan-Kettering Cancer Center
CV: coefficient of variation
FDR: false discovery rate
