# Supplementary material for: Plasma exosomal miRNAs-based prognosis in metastatic kidney cancer
Source: Oncotarget. 2017 Jul 22;8(38):63703–14. doi: 10.18632/oncotarget.19476 (PMC5609954; doi:10.18632/oncotarget.19476)
Supplement: Supplementary file 1 [file oncotarget-08-63703-s001.pdf]

Plasma exosomal miRNAs-based prognosis in metastatic kidney cancer

SUPPLEMENTARY MATERIALS

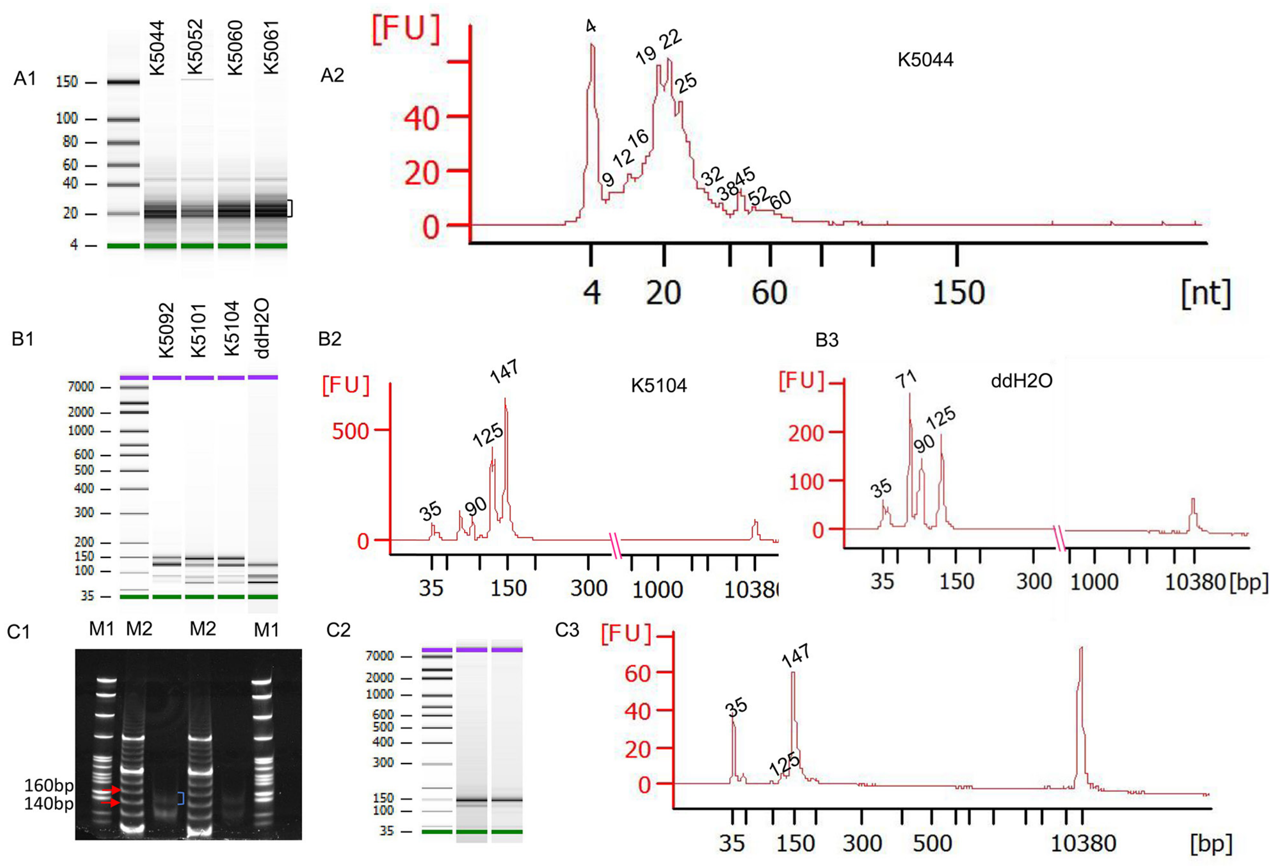

Supplementary Figure 1: Quality control of exosomal RNA libraries. (A1) Small RNA Chip shows exosomal RNA distribution. The range of miRNA is shown by the black square. **(A2)** An example of RNA peaks extracted from mRCC plasma exosome. **(B1)** High Sensitivity DNA Chip shows the size of the constructed miRNA libraries. **(B2)** An example of DNA peaks from one constructed RNA library. The 147 bp band corresponds to miRNAs, 125 bp band represents adaptor ligation products. **(B3)** Adaptor ligation product from ddH20 negative control. **(C1)** PAGE gel shows the pooled exosomal RNA sequencing libraries. The blue square frame represents the miRNA libraries recovered by gel size selection. M1: Molecular marker pBR322/MSP1, M2: Molecular marker O'RangeRuler 20 bp DNA ladder. **(C2)** Agilent Chip shows the recovered miRNA library pool. **(C3)** Final miRNA library pool for sequencing.

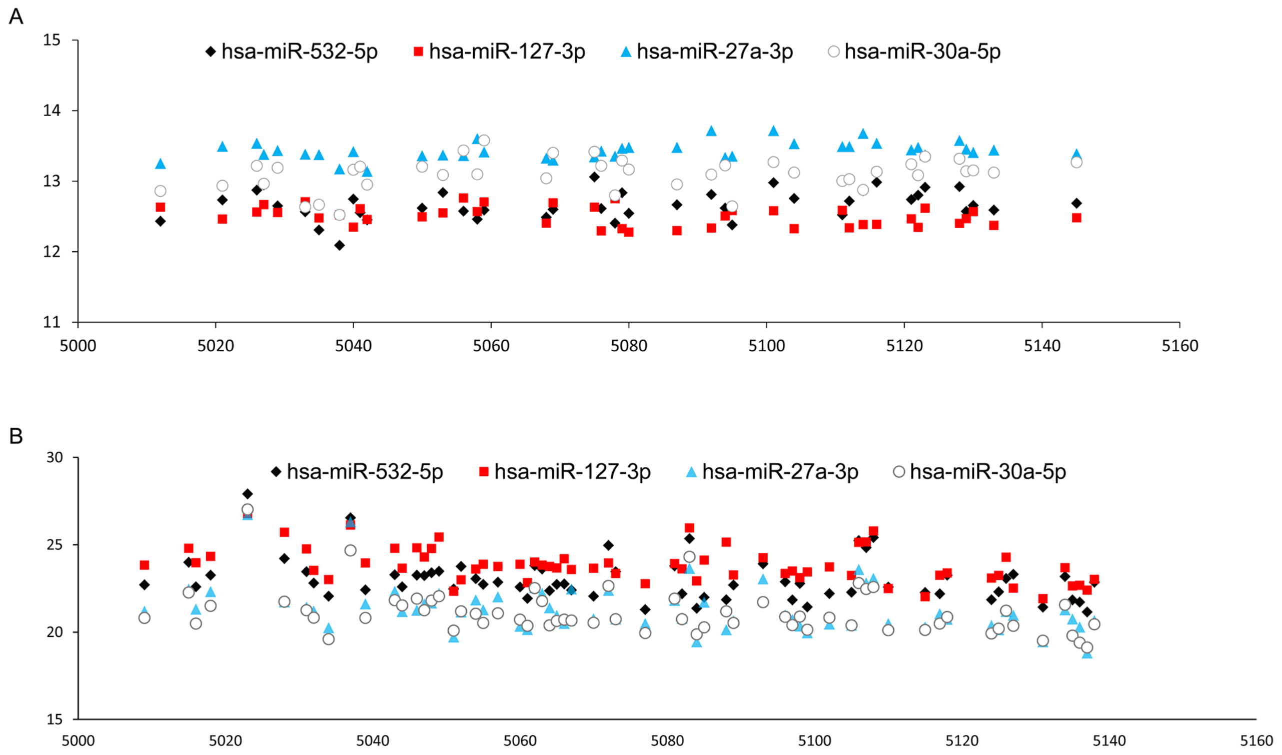

Supplementary Figure 2: The abundance and stability of candidate miRNAs for endogenous normalization controls. **(A)** Sequencing data from screening cohort of 44 samples. **(B)** qRT-PCR data from follow up cohort of 65 samples.

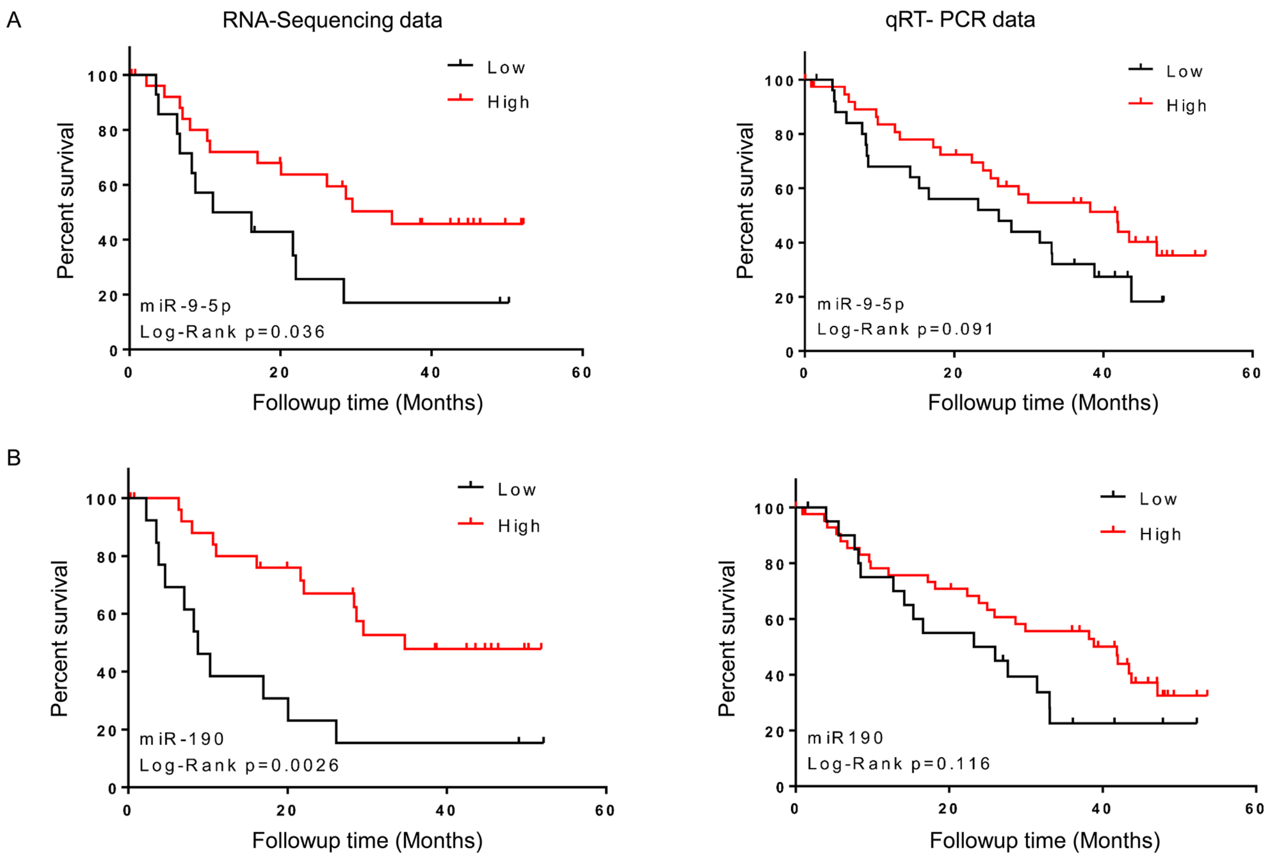

Supplementary Figure 3: Kaplan–Meier curves showing the association of miRNA abundance and OS in plasma of mRCC patients. Left panel: sequencing data from screening cohort. Right panel: qRT-PCR data from follow-up cohort. **(A)** miR-9-5p. **(B)** miR-190b.

Supplementary Table 1: Composition and distribution of each RNA species in the 44 RNA libraries.

See Supplemantery File 1

Supplementary Table 2: 322 read count >8 microRNA species identified in this study.

See Supplemantery File 2

Supplementary Table 3: The stability of 50 known microRNAs determined by Reffinder.

See Supplemantery File 3

Supplementary Table 4: Univariate hazard ratio analysis of clinical parameters and overall survival

		No pts.	HR	CI		p value
**Age**	>=65	32	0.80	0.58	2.01	0.8049
	<65	33				
**Gender**	Male	48	1.39	0.70	3.02	0.3624
	Female	17				
**Furhman grade**	>=3 vs rest	33	0.91	0.49	1.68	0.7526
	<=2	21				
**T stage**	>=T2	47	1.49	0.63	4.40	0.3902
	T1	9				
**N stage**	N1	10	1.82	0.71	4.12	0.1963
	N0	35				
**M stage at diagnosis**	M1	34	1.00	0.53	1.86	0.9982
	M0	30				
**Sarcomatoid differentiation**	Present	3	1.27	0.21	4.16	0.7511
	Absent	62				
**Coagulative necrosis**	Present	31	0.82	0.43	1.51	0.5178
	Absent	34				
**Total lines of systemic therapy**	>=2	28	1.11	0.55	2.36	0.7702
	<=1	24				
**MSKCC**	Poor vs good plus intermediate		2.02	0.94	6.81	0.0675
Good (n = 38)	Poor vs good		2.14	0.89	4.64	0.0371
Intermediate (n = 16)	Intermediate vs good		1.47	0.68	2.99	0.1096
Poor (n = 11)	Poor vs intermediate		1.45	0.56	3.63	0.1151
